# Health Policies, Physician Incentives, and Service Utilization for Non-Acute Diseases in Taiwan: The Case of Cataracts

**DOI:** 10.3390/healthcare13060587

**Published:** 2025-03-07

**Authors:** Yung-Hsiang Ying, Han-Chih Cheng, Mei-Jung Chen, Wen-Li Lee, Koyin Chang

**Affiliations:** 1 College of Management, National Taiwan Normal University, Taipei 106, Taiwan; 2 Department of Ophthalmology, Taipei Tzu Chi Hospital, New Taipei City 231, Taiwan; 3 Department of Biomedical Engineering, Ming Chuan University, Taoyuan 333, Taiwan; 4 Department of Healthcare Information and Management, Ming Chuan University, Taoyuan 333, Taiwan

**Keywords:** prospective payment system, cost sharing, national health insurance claim data, healthcare demand elasticity, non-acute disease

## Abstract

**Background:** Existing research highlights the necessity of tailoring cost-containment policies to specific treatments due to the varying benefits across different diseases. This study contributes additional insights by examining the impact of such policies on a non-acute condition—cataracts. **Methods:** Leveraging 16 years of national health insurance claim data, this research assesses the influence of three prevalent cost-containment payment schemes on healthcare service utilization. Outcome variables for analysis include the decision to adopt intraocular lens (IOL) insertion, outpatient visit volume, and healthcare expenditures. The robustness of the findings is enhanced through the use of statistical methods, such as logit, Poisson, negative binomial, and panel fixed-effect models. **Results:** Global budgeting reduces the likelihood of procedure adoption and negatively impacts the volume of outpatient consultation services. Cost sharing does not affect procedure adoption but significantly impacts outpatient service volume. The prospective payment scheme for cataract IOL treatment shows no long-term effects on service utilization, with treatment rates stabilizing after a few years of policy implementation. Despite reimbursement points remaining unchanged for over two decades, there is no evidence of the under-provision of treatment. **Conclusions:** This study underscores the significant responsiveness of both patients and providers to policy reforms in the non-acute disease category. Manipulating payment schemes can lead to cost savings, particularly when treatment plans and procedures exhibit increased elasticity in their provision.

## 1. Introduction

To mitigate the potential over-utilization incentivized by the traditional fee-for-service (FFS) payment model [1], countries worldwide have implemented various cost-containment measures to control rising healthcare expenses and alleviate workforce burdens, including patient cost sharing, global budgeting (GB), a type of insurance scheme that sets an upper limit on total spending. The prospective payment system (PPS) is a reimbursement method where health insurance payments are made based on a predetermined, fixed amount determined by disease category, such as diagnostic-related groups (DRGs)), and the prospective payment system (PPS) [2]. Since the introduction of Taiwan’s National Health Insurance (NHI) in 1995, these policies have been gradually implemented. Patient cost sharing reduces NHI expenditures by curbing patient demand for services. GB establishes an annual reimbursement cap for health insurance, while the PPS reimburses providers at fixed rates for specific surgical and inpatient care, ensuring consistent pricing for similar services across providers [1]. GB and the PPS are technical strategies for managing healthcare costs and reimbursement from insurers. While past studies mainly address short-term cost savings—often focusing on reduced hospital lengths of stay (LOSs)—research on the PPS’s long-term impact on outpatient services is limited. Acute conditions, such as cardiac events, may see minimal impact due to the critical nature of these services [3,4]. As a result, the true impact of health insurance policies may not be accurately assessed using acute conditions as research targets. To address this limitation and bridge the gap in the existing literature, this study examines the long-term effects of these policies on cataracts—a non-acute condition—by analyzing decisions to undergo procedures, the utilization of outpatient services, and overall healthcare expenditures, an area that remains underexplored. With growing emphasis on health-related quality of life and equity, chronic conditions with the potential for severe outcomes, such as vision loss, warrant significant attention and informed policy considerations.

Prospective payment systems (PPSs), designed to ensure quality patient care while controlling costs, are a form of bundled payment system [5,6]. These systems can raise issues like “cream skimming”, where providers may selectively treat healthier patients, potentially leaving critically ill patients without adequate care [7]. Under diagnosis-related group (DRG) coding, long-term disease-specific trends must be adjusted for patient comorbidities and other individual risk factors to accurately monitor service utilization and assess changes in provider behavior under the NHI-regulated incentive systems [8].

This research aims to examine behavioral responses to policy changes from both the demand and supply sides, focusing on cataract patients under a case-based payment system. Notably, this study is the first to specifically investigate a chronic, non-acute condition in this context. Cataracts were chosen as the research focus for several reasons. Firstly, they are prevalent in aging populations and progress gradually, impairing vision and quality of life if left untreated, with the potential to cause blindness [9]. Treatments include eye drops to slow progression and surgical intraocular lens (IOL) replacement. Patients may often live with cataracts for years before surgery becomes necessary. Secondly, for chronic conditions like cataracts, the high clinical standards required for NHI reimbursement may deter patients from seeking early intervention. Physicians, on the other hand, may adjust the content of their consultations based on these policies. Thirdly, cataract treatments, which primarily aim to improve quality of life, can be managed with minimal post-surgical care in outpatient settings. With this attribute of cataracts, we can better assess how payment policy reforms influence non-urgent healthcare service utilization without confounding complications.

This study is structured as follows: The next section provides a brief background on Taiwan’s current health policies and the relevant literature. Section 3 outlines the research methodology, data sources, and empirical strategies. This is followed by the presentation of research results and discussions. Finally, the conclusion summarizes the study’s findings and explores future research directions.

## 2. Background

### 2.1. Policy Changes of Taiwan NHI

Taiwan’s National Health Insurance (NHI) is a mandatory, government-run single-payer healthcare system that covers approximately 99.6% of legal residents. Service providers under NHI are categorized into four accreditation levels based on service type, function, and quality of care. To contain the rising healthcare costs, Taiwan has implemented the following three primary policy mechanisms:

#### 2.1.1. Cost Sharing

Cost sharing under NHI includes copayments set by NHI and registration fees determined by individual providers, structured according to service type. Copayment adjustments have been applied to inpatient, ambulatory, referral and non-referral outpatient services, prescription medications, lab tests, and high-user surcharge policies [5]. Each of these adjustments and reforms is detailed in Table 1.

#### 2.1.2. Prospective Payment Systems (PPSs)

Two PPSs currently operate in Taiwan’s NHI: case payment and Tw-DRGs. Case payment, introduced in October 1997, aimed to control healthcare costs while maintaining care quality, gradually increasing its share of total NHI reimbursements to 15.19% by 2001. Tw-DRGs were introduced in two phases starting in January 2010 and are mainly for hospital and inpatient services. Reimbursement revisions under the PPS have included adjustments in 1998, 2001, and 2007 [11]. For cataract patients, intraocular lens (IOL) treatments remain under case payment, where reimbursement rates have remained unchanged for two decades, unlike other treatments like laparoscopic cholecystectomy, which has seen frequent fee adjustments [12].

#### 2.1.3. Global Budgeting (GB)

The Global Budgeting (GB) system is another mechanism Taiwan’s NHIA uses to cap healthcare costs, limiting annual health insurance reimbursements to a predetermined amount. This budget was initially implemented for dental services in 1998 and was later extended to clinical (ambulatory) services in 2001 and hospital services in 2002. GB policies allow physicians to increase income by increasing service volumes within the capped budget, using a floating reimbursement rate that automatically adjusts to ensure expenditures do not exceed budgetary limits [13,14,15,16]. The target for one point is approximately 1 New Taiwan dollar (TWD), generally fluctuating between TWD 0.80–TWD 0.95 (with USD 1 = TWD 30.5 as of 2015). This downward adjustment ensures total payments stay within each quarter’s budget [17,18].

This study focuses on IOL insertion services for cataracts, as these policy structures could influence service utilization from the perspectives of both the demand and supply sides. Accordingly, policy indicators and fixed-time effects are included as control variables in the analysis models to measure supply-side influences on healthcare access.

### 2.2. Related Studies and Literature Gap

Research on healthcare cost containment focuses on three main areas.

First, patient cost sharing has been a central topic of discussion since the RAND experiments nearly half a century ago [19]. A moral hazard is an economic term that describes a situation where an economic actor has an incentive to increase its exposure to risk because it does not bear the full costs associated with that risk, particularly when insurance protection is in place. The concept originates from the insurance industry and refers to the demand response to changes in out-of-pocket costs [20,21]. Increased patient cost sharing can reduce moral hazard [22], though it may also risk compromising the quality of patient care [23]. Second, the implementation of the prospective payment system (PPS) has significantly impacted the hospital industry and how physicians and patients utilize hospital services [24]. Key components of the PPS include the use of administered prices instead of market forces, national base rates rather than hospital-specific rates (reflecting a policy of equalizing rates instead of equalizing pressure), and per-case payments rather than payments per day, service, or procedure. Proponents of the PPS anticipated that reduced spending would align with payment reductions through shorter lengths of stay (LOSs), reduced care intensity, and more efficient hospital operations. Most research on expenditures supports these expectations, identifying reduced LOSs, care intensity, and improved efficiency as sources of cost reductions. However, many studies focus on the initial years of PPS implementation, when LOS reductions were most pronounced, and financial pressures were relatively low [25]. The evidence indicates that the PPS improved clinical and general hospital productivity, with the potential for continued improvement [26]. For example, Long et al. (1987) found that clinical productivity improved significantly in the first year of the PPS due to substantial reductions in inputs per unit of output across 49 DRGs. They also observed that the most significant effects occurred during the initial year, with a diminished impact in subsequent years, attributing early changes to a one-time reduction in LOSs [27]. Lastly, patient mortality outcomes and facility profit margins are closely related to facility size, with smaller facilities being particularly vulnerable to fixed payment rates [24,28].

Numerous studies utilizing Taiwan’s data have yielded similar findings. Research on case payment systems for procedures, such as septomeatoplasty and laryngo-microsurgery [29], cataract intraocular lens (IOL) insertion [30], cesarean section delivery [31], knee replacement [32], and fistulectomy [33], has provided valuable insights. Generally, these studies focus on the short-term effects of the PPS, concluding that the average length of stay was significantly reduced, total claimed hospitalization costs decreased notably, and outpatient visits increased substantially for specific conditions. Another strand of research focuses on hospital service utilization before and after policy changes under the GB system; however, it does not quantify changes in service utilization at the patient level within a specific disease category [34]. Few studies, if any, have examined the long-term effects of the PPS. To address this gap, we analyzed 16 years of data on cataract patients for whom IOL insertion is the standard treatment under Taiwan’s National Health Insurance Administration (NHIA) case payment scheme. By isolating the effects of complications associated with inpatient services, this study aims to provide a comprehensive assessment of the PPS’s long-term impact across multiple dimensions.

## 3. Methodology

The extensive literature analyzing the impacts of health policy reforms often employs methods such as ANOVA, chi-square testing, and difference-in-differences (DID) using single-year or short-term claims data. In this study, we focus on cataract patients, leveraging 16 years of longitudinal data. We hypothesize that controlling for serial payment scheme changes across different years is essential for fully understanding variations in healthcare utilization for non-urgent care.

In medical practice, the development of treatment guidelines by clinicians typically requires a prolonged period. Consequently, policy-induced changes in practice may only produce short-term effects [35]. Given the frequent policy changes in Taiwan, longitudinal data provide critical insights into the impacts of these reforms.

### 3.1. Data Sources

The data for this study were obtained from the Ministry of Health and Welfare (MOHW) databank, which houses National Health Insurance (NHI) claims data. Taiwan’s NHI database is among the largest nationwide population-based databases globally and has been widely tested and validated in scientific research [36]. Additionally, reimbursement points data (GB points) were collected from various sources available on the MOHW’s official website [37].

With the NHI program covering over 99.5% of Taiwan’s legal residents, it represents the most comprehensive healthcare database in the country. In 2010, two million beneficiaries were randomly sampled from the entire population, and their claim information was traced back to 2000 and forward to 2017, creating a robust longitudinal dataset. For this study, we utilized data up to 2015 to avoid inconsistencies introduced by the transition from ICD-9 to ICD-10 coding.

Each outpatient visit’s primary diagnosis in the NHI database is coded based on ICD-9. Monthly claims summaries for outpatient and inpatient services were extracted from datasets containing two million randomly sampled patients (HEALTH-01: H_NHI_OPDTE and HEALTH-02: H_NHI_IPDTE). Additional data on beneficiaries’ backgrounds, including registration, demographics, and economic information, were sourced from HEALTH-07: H_NHI_ENROL. Institutional and physician characteristics were obtained from HEALTH-25: H_NHI_MEDFA and HEALTH-29: H_DOH_MEDPE, respectively.

The outcome variables for this study include annual patient-level data on the number of outpatient services for cataract diagnoses (ICD-9: 366–367), intraocular lens (IOL) insertions (ICD-9-OPM: 13.x) during ophthalmology visits, and total healthcare expenditure (measured in points).

### 3.2. Empirical Strategies

The empirical analyses consist of three components: (1) modeling the decision to undergo the IOL procedure using a panel logit model, (2) analyzing the volume of outpatient service utilization with panel Poisson and negative binomial models, and (3) examining health service expenditures through a panel fixed-effects OLS regression. The details of each approach are outlined below:

(1) Decision to Undertake the IOL Procedure (A Logit Model)

For cataracts, some patients underwent the treatment procedures through IOL insertion (categorized as Case_Type 81 or C1 in NHI database coding), while the majority of the patients did not. For the binary outcome variable, we adopted the most employed logistic regression model, or logit [38], for the individual patient data, as follows:

(1) $$\mathrm{Pr}\left(IOL=1|X_{it\;}{,H}_{it}\right)=\Phi (\beta_{0}+{\beta_{1}{Policy}_{it}+\beta }_{2}{\;X}_{it}+\beta_{3}H_{it}+{\beta_{4}Dist}_{t}+\beta_{5}Time+\varepsilon_{pt})$$

where Φ takes the form of the logistic distribution functions. The dependent variable, IOL, is one if the patient undertook IOL surgery and is zero otherwise. Policy is a vector of independent variables that capture national health insurance policy changes, including copayment and reimbursement points, which are both regulated by the NHIA. $X_{it}$ are the demographic characteristics of patient *i* in time t. $H_{it}\;$ represents the characteristics of the healthcare institution in which patient *i* receives his/her services, including the Herfindahl and Hirschman Index (HHI) for the competitiveness of the institution, age, the scale of the institution measured by the number of physicians working full-time in the institution, and the accreditation tier of the institutions. Time represents the time-fixed effect that captures the dynamic changes in the general economic condition and medical practice norm over time, and Dist represents the district-specific fixed effects;

(2) Utilization of Outpatient Services (Poisson and Negative Binomial Models)

To better understand cataract patients’ utilization of healthcare services beyond their decision to undergo the IOL procedure, we also examined how health policies and institutional characteristics influence changes in the volume of outpatient visits. Given that the number of physician office visits per patient is count data, Poisson and negative binomial regression models were more appropriate for this analysis [18,39]. Following the analytical framework of Chandra et al. (2010) [23], the quasi-experimental change in the NHI copayment policy was modeled as follows:

(2) $${Serv}_{pt}=\beta_{0}+{\beta_{1}{IOL}_{it}+\beta_{2}{Policy}_{it}+\beta }_{3}{\;X}_{it}+\beta_{4}H_{it}+{\beta_{5}Dist}_{t}+\beta_{6}Time+\varepsilon_{pt}$$

where *Serv* is the number of outpatient visits of patient i in time t. IOL is a binary variable indicating whether or not the patient undertakes the IOL insertion under the case payment scheme. The effect of the IOL procedure is captured by β1, which quantifies the additional number of physician office visits required when patients undergo the IOL procedure under the case payment scheme. The remaining covariates are consistent with those described in the previous section. The regression coefficients are denoted as β, and ε_pt_ is i.i.d with normal properties, i.e., $\sim N(0,\sigma^{2})$;

(3) Annual Cataract-Related Treatment Expenditure (Fixed Effect and 2SLS Methods)

To gain a comprehensive understanding of how patients utilize healthcare services, we analyzed the total health expenditure for cataract-related treatments annually. The dependent variable is the annual reimbursement points claimed under the National Health Insurance program, combined with each patient’s copayment. The fixed-effects model used in this analysis follows a structure similar to Equation (2), with the dependent variable replaced by the logarithm of expenditure:

(3) $$Expditure=\beta_{0}+{\beta_{1}IOL+\beta_{2}{Policy}_{it}+\beta }_{3}{\;X}_{it}+\beta_{4}H_{it}+{\beta_{5}Dist}_{t}+\beta_{6}Time+\lambda_{t}+\varepsilon_{pt}$$

Given that the decision to undergo the IOL procedure could be endogenous and influenced by policy and institutional characteristics, a two-stage least squares (2SLS) method was applied as an alternative for comparison [40]. This equation was then decomposed into two stages, as outlined below:

$$Expditure=\beta_{0}+{\beta_{1}IOL+\beta_{2}{Policy}_{it}+\beta }_{3}{\;X}_{it}+\beta_{4}Time+\varepsilon_{it}\;IOL=a_{0}+a_{1}{Policy}_{it}+a_{2}H_{it}+{a_{3}Dist}_{t}+a_{4}Time+v_{it}$$

where **υ**_it_ and **ε**_it_ are zero–mean error terms, and their correlation is assumed to be non-zero.

## 4. Results

**Figure 1:**
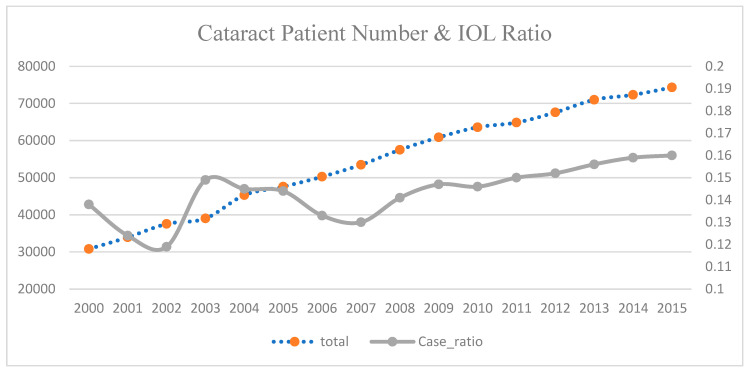
Total cataract case number and percentage of cataract patients using IOL procedure.

We identified patients with cataracts and related eye diseases from the two-million-population database provided by the NHIA, administered by the MOHW. Among these patients, a subset underwent the IOL insertion procedure. Although the number of IOL cases has been trending upward, the proportion of cataract patients undergoing the procedure has increased only marginally, suggesting that cataract treatment practices have remained relatively stable over time, as shown in Figure 1.

Table 2 and Table 3 present a statistical summary of patients by gender, treatment type, and employment. The screening results indicate that more patients are female (59%), but male patients have more outpatient visits, on average, and are more likely to undergo the IOL procedure (13.5% of males compared to 12.7% of females). Patients who underwent the IOL procedure had an average of 2.5 more outpatient visits than those who did not.

In terms of employment, Table 3 shows that patients working in the agricultural sector have the highest ratio of IOL procedures and medical expenditures. Patients who work as independent contractors report the highest number of physician office visits.

A statistical summary by year is provided in Table 4, with notable observations as follows:

First, a sharp upward trend in the number of cataract patients is observed over the study period. The incidence rate rose from 1.5% in 2000 to 3.71% in 2015, more than doubling during this period. Second, per capita medical expenditure increased from TWD 5560.1 in 2000 to TWD 6390 in 2015 (nominal figures, unadjusted for inflation). However, when adjusted for the Consumer Price Index (CPI), the 2015 figure is equivalent to TWD 5516.37 in 2000 terms, indicating a slight decrease in real expenditure over 15 years.

(1) Decision to Adopt IOL

The results of the logit model are presented in column (1) of Table 5, where odds ratios are reported. The covariate “Point,” representing the impact of global budgeting, shows an odds ratio significantly below one, indicating that healthcare providers may cautiously limit procedures to preserve a higher NHIA reimbursement point balance.

The odds ratio for copayment is statistically insignificant and close to 1, suggesting that patients’ decisions to undergo the procedure are not influenced by the copayment amount. Male patients are 1.13 times more likely to undergo the procedure than female patients. Additionally, income and the Charlson Comorbidity Index (CCI) negatively affect the likelihood of undergoing the procedure, while premium levels have a positive effect. The regression results for employment categories align with the descriptive statistical analyses. Patients working in the agricultural sector have the highest odds of undergoing the procedure, whereas independent contractors have the lowest odds.

Regarding institutional characteristics, higher Herfindahl–Hirschman Index (HHI) values, being treated at a medical center, and older institutional age are associated with a higher likelihood of the procedure being ordered for patients;

(2) Number of Outpatient Service Volume

Both Poisson and negative binomial analyses yield comparable results. The incidence rate ratio (IRR) is presented in Columns (2) and (3) of Table 5. Consistent with the IOL decision model, the number of outpatient services is negatively affected by the “Point” covariate. However, unlike the previous model, the copayment amount is also negatively associated with outpatient service utilization, suggesting that patients have some control over their decisions to visit physicians’ offices. Patients who undergo the IOL procedure have a 2.07 IRR compared to those who do not undergo the procedure, indicating a significantly higher volume of outpatient visits for the IOL group. Male patients, older patients, and those in better health tend to make fewer outpatient visits compared to female, younger, and less healthy patients. Regarding institutional characteristics, larger institutions and those with greater market power are more likely to induce higher volumes of outpatient services;

(3) Medical Expenditure

After experimenting with both panel OLS and 2SLS methods, as shown in Columns (5) and (6) of Table 5, respectively, the 2SLS method produces more robust results in terms of the significance levels of the covariates. Therefore, the interpretation of the results focuses on the 2SLS model. The dependent variable is in its natural logarithmic form, so the reported coefficients of the covariates represent the percentage change in medical expenditure. As observed, an increase in copayment, as regulated by the NHIA, leads to a rise in medical expenditure. Each adjustment in copayment corresponds to an approximate 10.3% increase in total expenditure. Patients who undergo the IOL procedure incur, on average, 12.22 times higher medical expenditure than those who do not undergo the procedure. For patient characteristics, age and the Charlson Comorbidity Index (CCI) significantly impact expenditures. Older and less healthy patients tend to spend more than their younger and healthier counterparts. Regarding institutional characteristics, market power (HHI) and the age of the institution have a negative effect on medical expenditure, while being a medical center and larger institutional size positively influence patient expenditures;

(4) Robustness with Propensity Score Matching (PSM) Analyses

Some extreme, non-representative participants in the sample could distort the effects of each covariate on the outcome variables of interest. In the case of cataracts, the patients of interest are those who are more severely affected, either having already received the IOL procedure or being on the verge of receiving it. To address this, matching patients with similar backgrounds and characteristics from both groups helps balance the confounders and reduces bias in the comparison. This approach allows for a more accurate estimate of the true treatment effect.

**Figure 2:**
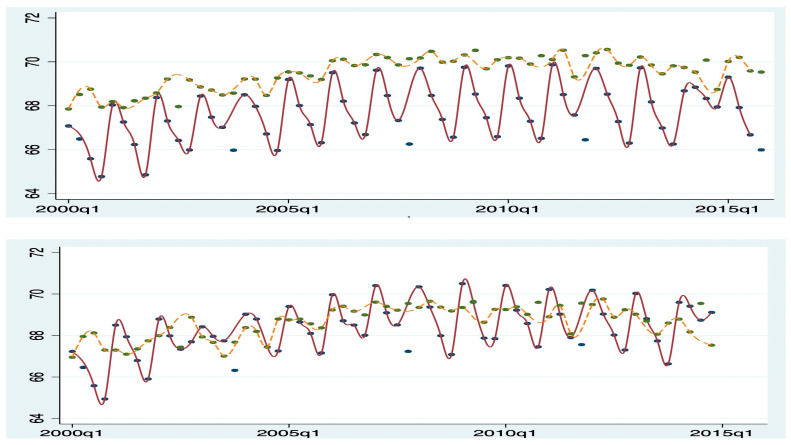
Quarterly patient average age before and after PSM. Note: dotted line: with IOL; solid line: no IOL. Top: All sampled patients; bottom: matched patients only.

**Figure 3:**
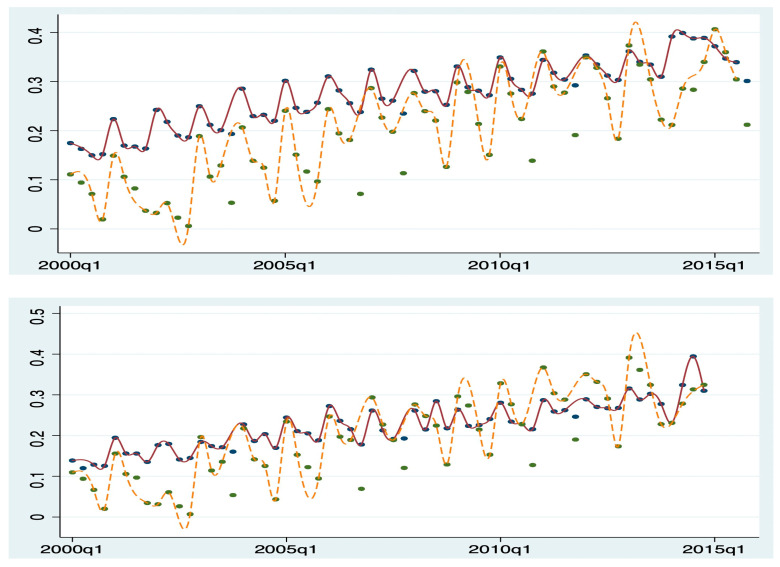
Quarterly patient average CCI before and after PSM. Note: dotted line: with IOL; solid line: no IOL. Top: all sampled patients; bottom: matched patients only.

For instance, in Figure 2 and Figure 3, the upper panels display the patients’ age and CCI before applying the matching method, while the lower panels show the sample means after matching. It is evident that patients in the IOL group are older and healthier. However, after using propensity score matching (PSM), the covariates between the two groups become more balanced. Diagnostic balance testing of the PSM suggests that matching based on the estimated propensity score effectively balanced the covariates. This testing is not reported here for space reasons but is available upon request.

The regression analyses were then rerun using the matched sample, which included 165,764 observations. The results from these regression analyses are presented in Table 6. Overall, the results are comparable to those in Table 5, but the coefficients are generally more statistically significant. A key difference is that the odds ratio for “Point” in Model (1) is much smaller, indicating a stronger response from the supply side when the global budget reimbursement rate is adjusted in deciding whether or not to undertake the IOL procedure. In Models (2) and (3), the IRR for “Point” exceeds 1, while “Income” and “Premium” become statistically insignificant. This suggests that outpatient visits are more influenced by policy changes than by patients’ income levels. Lastly, the odds ratio for the IOL procedure is considerably smaller in Models (2) and (3) of Table 6 compared to Table 5. By comparing patients between the matched groups, the treatment effect is more accurately estimated.

## 5. Discussions

Various cost-containment strategies have been implemented by health insurance agencies worldwide to curb rising healthcare expenditures and alleviate workforce burden. Taiwan introduced a case payment policy in October 1997 for selected diseases, with short-term effects observed, primarily through savings resulting from reduced inpatient length of stay (LOS) [11]. However, the impact on outpatient services remains unclear. This study focuses on patients with cataracts, aiming to explore their outpatient service-seeking behavior under the prospective payment system (PPS). Specifically, the study examines factors such as the decision to undertake the IOL procedure, outpatient visit volume, and treatment expenditures. The study also aims to provide a clear understanding of the effects of policy changes on chronic, non-acute diseases.

Our data, sourced from the Taiwan NHIA, show an upward trend in the number of patients with cataract-related diseases. However, the ratio of IOL procedures to cataract patients remained relatively stable between 2000 and 2007. Health policy changes, along with the impact of SARS in 2002, appear to influence the adoption of the IOL procedure. This research analyzes the effects of policy changes in three key areas. Global budgeting sets a financial ceiling for outpatient expenditures while fostering competition among healthcare providers by adjusting the reimbursement “point” to meet the global budget constraint.

### 5.1. Policy Implications

Our analyses reveal that the reimbursement point significantly negatively affects the decision to undergo the IOL procedure, consistent with previous research on global budgeting [35,41]. Specifically, the results suggest that patients are approximately three times less likely to receive an IOL procedure with a 10% increase in reimbursement points (Model (1) of Table 6). When comparing the number of outpatient visits between unmatched and matched data, the effect of the reimbursement point turns positive in the matched data. A plausible explanation is that the matched cohort consists of patients with a critical need for the IOL procedure, who also have an urgent need for physician consultations. Therefore, when the reimbursement point is higher, physicians are more likely to encourage these patients to return for additional visits.

The policy of patient cost sharing consistently demonstrates a negative impact on the number of outpatient visits. Specifically, for every 1% increase in cost sharing, the number of outpatient visits decreases by approximately 4.1% to 4.8%, indicating a more elastic response than suggested in the previous literature [42]. For patients who undergo the IOL procedure, the number of outpatient visits increases by 37.5% to 42.7%, while expenditures rise between 3.31 and 15.84 times compared to patients who do not undergo the procedure.

### 5.2. Impacts of Demographics and Facility Characteristics

Regarding demographic characteristics, the results are consistent with the existing literature [43]. Female patients are more likely than their male counterparts to seek outpatient services, but male patients are more likely to undergo the IOL procedure. Older patients are more likely to undergo the IOL procedure but tend to have fewer outpatient visits, resulting in lower cataract-related healthcare expenditures. Less healthy patients are less likely to undergo the IOL procedure, but they tend to have more outpatient visits and higher healthcare expenditures compared to healthier patients. Wealthier patients are less likely to undergo the IOL procedure, likely because they have the resources to better manage their eye health, such as through protective sunglasses or dietary supplements that may delay cataract progression. Additionally, they might opt for alternative treatments requiring out-of-pocket payments not covered by National Health Insurance (NHI) [44].

In terms of institutional characteristics, a significant supplier-induced demand is observed. Facilities with more employed physicians or higher market power are more likely to order the IOL treatment and exhibit greater outpatient service volume, leading to higher expenditures for patients. These effects are statistically significant at the 1% level. Additionally, facilities with longer operational histories are also more likely to order the IOL procedures, though the impact on outpatient service volume and revenue requires further investigation.

### 5.3. Limitations

There are several limitations to this study. First, the NHIA claims data are available only from 2000 onward, which is a few years after the implementation of the case payment scheme for cataracts. This means there is no pre-period data for comparison. Second, the increasing number of cataract patients could be attributed to external factors, such as increased screen time and greater public awareness of the disease. Additionally, advancements in medical technology may have contributed to the higher volume of treatments due to the effectiveness of the IOL procedure. As a result, this study does not fully capture the impact of the PPS scheme on the trade-off between a shortened inpatient length of stay and an increased outpatient service volume. Additionally, the findings on cataract care may not be generalizable to other disease types. Furthermore, the study period, which spans from 2000 to 2015, may limit the applicability of the results to more recent policy impacts.

## 6. Conclusions

Cataracts are responsible for approximately 50% of blindness worldwide [8]. However, due to their non-urgent nature and lack of immediate life-threatening implications, healthcare providers may exhibit flexibility in offering treatment and consultations, particularly in response to changes in health reimbursement policies. This elastic response can result in treatment delays or the absence of treatment altogether. This study investigates the long-term effects of three cost-containment policies using Taiwan NHI outpatient claim data for cataract patients, focusing on treatment decisions, outpatient service volume, and healthcare expenditures. Unlike most previous studies, which predominantly use short-term data to analyze the impacts of health policies, this research spans 16 years, providing insights into the enduring effects of these policies. Additionally, by employing the PSM method to exclude patients with extreme characteristics and focus only on individuals with similar demographic and clinical backgrounds, this study provides robust insights into the local treatment effects of healthcare policies.

This study highlights the importance of tailoring cost-containment policies based on treatment value and urgency to unlock potential healthcare cost savings. The findings suggest that global budgeting reduces the likelihood of procedure adoption and negatively impacts outpatient services, primarily influencing provider behavior through reimbursement rates. This policy significantly affects both the decision to perform the IOL procedure and outpatient service volume. In contrast, cost-sharing impacts outpatient service volume without affecting procedure adoption.

The findings also indicate the presence of supplier-induced demand (SID). Institutions with greater market power and a higher number of employed physicians are more likely to recommend the IOL procedure and generate higher outpatient service volumes. These policy impacts underscore the profound influence of reimbursement structures on both patient care-seeking behavior and provider practice patterns, particularly for non-acute conditions such as cataracts.

To optimize healthcare delivery for non-urgent conditions, the value of treatment should be determined through rigorous professional medical judgment rather than patient preferences alone. Reimbursement policies serve as effective mechanisms to guide both providers and patients in utilizing healthcare services. Therefore, fee structures should be strategically adjusted to align provider and patient behaviors with evidence-based care standards. While it has long been assumed that modifications to reimbursement policies will produce straightforward and predictable impacts on utilization, this study contributes to the literature by demonstrating how healthcare actors could make predictable behavioral adjustments in response to these policy changes.

The results of this study provide valuable insights for future healthcare policy development, particularly in managing chronic, non-acute conditions. Further analyses could be beneficial if disease types were examined in greater detail across population groups, considering geographical variations and patients with different comorbidities. Such an approach could enhance the understanding of how chronic conditions interact with insurance payment schemes, leading to more effective policy adjustments.

## Figures and Tables

**Table 1:** Copayment changes for outpatient services and medications under Taiwan National Health Insurance.

Time	Medical Center	Regional Hospitals	District Hospitals	Clinics	Drug Fee	High-User Surcharge
May 1995	100	100	50	50	None	None
March 1997	150	100	50	50	None	None
August 1999	150	100	50	50	20~100	<50: TWD 0; 50~156: TWD 50; >156: TWD 100;
January 2000	150	100	50	50	20~100	<25: TWD 0; 25~156: TWD 50; >156: TWD 100;
July 2001	150	100	50	50	20~200	<25: TWD 0; 25~156: TWD 50; >156: TWD 100;
September 2002	210	140	50	50	20~200	<25: TWD 0; 25~156: TWD 50; >156: TWD 100;
January 2004	210	140	50	50	20~200	None
July 2005	360	240	80	50	20~200	None
April 2017	420	240	80	50	20~200	None
July 2023	420	240	80	50	10~300	None

Note: Some data are retrieved from NHIA webpage [10]. High user refers to a number of physician office visits that exceeded 50 times a year in 1999. This number decreases to 25 times after 2000. Medication copayment is charged by TWD 20 for each TWD 100 total drug fee incurred with a maximum TWD 100 (TWD 200 after 2000) surcharge in copayment. All dollar values are in TWD, which is approximately exchanged to USD at the rate of USD 1 to TWD 30.

**Table 2:** Summary statistics by gender and IOL treatment.

	ALL	NO IOL	IOL	FEMALE	MALE
					
Premium	832.2	835.0	813.8	847.9	810.1
	(1034.0)	(1036.5)	(1017.6)	(1025.5)	(1045.5)
					
Income	22,932.8	22,947.3	22,836.2	20,665.1	21,056.2
	(21,534.2)	(21,740.2)	(20,107.8)	(18,715.0)	(20,003.7)
					
Age	66.56	67.09	67.42	67.83	69.12
	(10.43)	(10.19)	(10.17)	(9.912)	(11.19)
					
Male (%)	0.419	0.417	0.432	0	1
	(0.493)	(0.493)	(0.495)		
					
Outp’t visit volume	3.577	3.249	5.765	3.52	3.613
	(3.610)	(3.415)	(4.078)	(3.50)	(2.770)
					
Expenditure	5883.4	2519.4	28,292.0	5688.9	5638.8
Average	(11,013.4)	(6078.0)	(10,279.9)	(10,722.7)	(10,149.8)
					
CCI	0.293	0.299	0.251	0.269	0.32
	(0.700)	(0.704)	(0.669)	(0.640)	(0.731)
					
IOL (%)	0.131	0	1	0.127	0.135
	(0.337)			(0.333)	(0.341)
					
Total P’t No.	870,065	756,500	113,565	505,900	364,165
					

**Table 3:** Summary statistics by employment type.

	CIVIL SERVANT	PUBLIC SECTOR	PRIVATE SECTOR	AG. WORKER	SCHOOL	INDEP. CONT	OTHER
							
Premium	1703.7	1680.0	1465.9	315.6	1078.7	443.1	1070.2
	(1251.7)	(1014.1)	(1491.4)	(198.8)	(707.5)	(604.2)	(767.2)
							
Income	48,411.4	56,832.7	40,029.4	20,676.5	35,292.8	1128.7	25,844.3
	(27,338.1)	(22,001.1)	(25,110.8)	(1318.9)	(15,108.9)	(109.6)	(12,861.8)
							
Age	68.66	66.85	65.36	70.82	65.68	71.48	63.35
	(10.60)	(11.25)	(11.08)	(8.672)	(11.23)	(9.791)	(10.34)
							
Male (%)	0.392	0.463	0.414	0.388	0.377	0.496	0.364
	(0.488)	(0.499)	(0.493)	(0.487)	(0.485)	(0.500)	(0.481)
							
Outp’t	3.631	3.519	3.439	3.527	3.434	3.840	3.417
visit volume	(3.599)	(3.816)	(3.515)	(3.527)	(3.605)	(3.803)	(3.523)
							
Expenditure	5415.1	5504.0	5757.0	6510.8	5512.9	5652.5	5596.6
Average	(10,416.6)	(10,588.0)	(10,968.4)	(11,550.7)	(10,451.6)	(10,616.2)	(11,031.6)
							
CCI	0.272	0.274	0.312	0.248	0.303	0.309	0.324
	(0.675)	(0.681)	(0.718)	(0.654)	(0.707)	(0.715)	(0.730)
							
IOL (%)	0.113	0.118	0.125	0.161	0.119	0.114	0.122
	(0.316)	(0.323)	(0.331)	(0.368)	(0.324)	(0.317)	(0.327)
							
Obs. No.	56,413	5776	191,396	238,578	14,173	226,675	137,054
							

**Table 4:** Summary statistics by year.

	2000	2001	2002	2003	2004	2005	2006	2007
								
Premium	597.1	621.8	659.8	724.3	740.9	756.8	782.1	800.6
	(672.5)	(705.8)	(771.8)	(872.3)	(891.0)	(926.5)	(973.5)	(995.5)
								
Income	18,550.0	18,730.2	19,299.1	20,597.0	20,665.1	21,056.2	21,485.1	22,138.8
	(13,900.2)	(14,484.5)	(16,003.6)	(18,631.4)	(18,715.0)	(20,003.7)	(21,125.3)	(21,480.4)
								
Age	66.56	67.09	67.42	67.71	67.86	68.31	68.59	68.65
	(10.43)	(10.19)	(10.17)	(10.43)	(10.51)	(10.11)	(10.15)	(10.44)
								
Male (%)	0.397	0.404	0.405	0.414	0.413	0.413	0.415	0.417
	(0.489)	(0.491)	(0.491)	(0.493)	(0.492)	(0.492)	(0.493)	(0.493)
								
No. of	3.342	3.392	3.435	3.400	3.567	3.613	3.620	3.609
Outp’t visits	(2.590)	(2.574)	(2.588)	(2.582)	(2.751)	(2.770)	(2.821)	(2.830)
								
Expenditure	5560.1	5775.5	5824.0	5703.4	5760.9	5638.8	5511.4	5486.2
Average	(10,614.9)	(10,934.0)	(10,645.5)	(10,321.4)	(10,383.7)	(10,149.8)	(9966.9)	(10,026.0)
								
CCI	0.152	0.179	0.209	0.210	0.238	0.257	0.272	0.279
	(0.532)	(0.572)	(0.612)	(0.607)	(0.640)	(0.661)	(0.678)	(0.685)
								
IOL %	0.138	0.124	0.0459	0.149	0.145	0.144	0.133	0.130
	(0.198)	(0.188)	(0.114)	(0.207)	(0.206)	(0.206)	(0.203)	(0.201)
								
Patient No.	30,796	33,943	37,547	39,036	45,293	47,586	50,266	53,491
	**2008**	**2009**	**2010**	**2011**	**2012**	**2013**	**2014**	**2015**
								
								
Premium	833.5	816.2	844.9	868.4	923.7	966.6	962.1	970.9
	(994.7)	(975.0)	(1060.7)	(1123.8)	(1163.4)	(1197.5)	(1174.1)	(1161.9)
								
Income	22,968.2	22,790.4	23,459.7	24,205.8	24,667.6	25,188.9	25,573.5	26,236.7
	(21,662.1)	(21,474.8)	(22,510.1)	(23,290.1)	(23,719.8)	(24,426.4)	(24,616.9)	(24,859.0)
								
Age	68.77	68.83	68.77	68.83	68.78	68.63	68.46	68.30
	(10.57)	(10.67)	(10.81)	(10.46)	(10.42)	(10.51)	(10.62)	(10.50)
								
Male (%)	0.418	0.424	0.425	0.419	0.416	0.456	0.413	0.414
	(0.493)	(0.494)	(0.494)	(0.493)	(0.493)	(0.498)	(0.492)	(0.493)
								
No. of	3.638	3.639	3.605	3.626	3.642	3.632	3.591	3.565
Outp’t visits	(2.873)	(2.897)	(2.917)	(2.941)	(2.958)	(2.944)	(2.911)	(2.879)
								
Expenditure	5676.4	5855.3	5808.3	6000.6	5989.9	6237.1	6171.1	6390.0
Average	(10,408.0)	(10,723.7)	(10,673.0)	(11,051.1)	(11,190.6)	(11,886.4)	(12,293.5)	(12,466.1)
								
CCI	0.286	0.297	0.311	0.321	0.331	0.343	0.390	0.351
	(0.692)	(0.699)	(0.717)	(0.726)	(0.736)	(0.745)	(0.790)	(0.748)
								
IOL (%)	0.141	0.147	0.146	0.150	0.152	0.156	0.159	0.160
	(0.213)	(0.219)	(0.221)	(0.227)	(0.230)	(0.232)	(0.0736)	(0.238)
								
	57,512	60,866	63,605	64,867	67,594	70,998	72,331	74,334

Note: Figures are yearly average. Standard errors are in parentheses. Premium and expenditure are in TWD. IOL indicates the percentage of intraocular lens procedure cases. Data are extracted from outpatient claims, thus inpatient procedures are not shown, resulting in a low average expenditure.

**Table 5:** Regression results—whole sample.

	(1)	(2)	(3)	(4)	(5)
	Logit	Poisson	NBinomial	OLS	2SLS
Policy Effects					
Point	0.688 ***	0.624 ***	0.593 ***	−0.32 ***	0.06
	(−3.30)	(−18.47)	(−24.58)	(−10.15)	(0.44)
Copay	1.003	0.982 ***	0.981 ***	0.01	0.103 ***
	(0.16)	(−5.02)	(−6.36)	(1.11)	(9.03)
IOL		2.078 ***	2.072 ***	3.27 ***	12.22 ***
		(294.25)	(346.00)	(1187.28)	(11.44)
Pt Chara.					
Male	1.129 ***	0.980 ***	0.993 *	−0.008 **	1
	(12.66)	(−7.19)	(−2.46)	(−2.44)	(.)
Age	1.008	0.955 ***	0.945 ***	−0.04 ***	0.41 *
	(0.99)	(−26.04)	(−33.27)	(−17.64)	(1.88)
Income	0.932 ***	0.996	0.990 ***	−0.01 *	0.008
	(−5.97)	(−1.21)	(−3.38)	(−1.68)	(0.36)
Premium	1.070 ***	0.992 ***	0.994 **	−0.000	−0.03 *
	(8.03)	(−3.74)	(−2.89)	(−0.31)	(−1.95)
CCI	0.878 ***	1.112 ***	1.121 ***	0.19 ***	0.26 ***
	(−18.96)	(70.24)	(88.08)	(81.03)	(18.92)
Employment					
Public Sector	1.062	0.966 **	0.977	−0.03 **	0.086
	(1.07)	(−2.33)	(−1.67)	(−1.69)	(0.79)
Private Sector	1.226 ***	0.968 ***	0.973 ***	−0.02 ***	−0.21
	(9.81)	(−5.97)	(−5.37)	(−2.57)	(−0.50)
Farm	1.554 ***	0.920 ***	0.911 ***	−0.039 ***	−0.641
	(18.53)	(−13.21)	(−15.66)	(−4.77)	(−1.00)
Education	1.172 ***	0.967 ***	0.971 ***	−0.023 *	−0.008
	(4.03)	(−3.21)	(−3.06)	(−1.77)	(−1.04)
Indep. Contract	0.810 ***	0.972 **	0.956 ***	−0.047 **	−0.002
	(−4.75)	(−2.41)	(−4.14)	(−3.07)	(−0.02)
Other	1.201 ***	0.964 ***	0.964 ***	−0.03 ***	−0.07
	(8.26)	(−6.38)	(−6.68)	(−3.62)	(−1.53)
Institution Ch.					
HHI	1.101 ***	1.015 ***	1.011 ***	0.035 ***	−0.119 ***
	(9.97)	(6.05)	(4.77)	(10.51)	(−4.65)
Med. Center	1.197 ***	1.019 ***	1.025 ***	0.05 ***	0.052 **
	(9.38)	(4.14)	(6.65)	(7.93)	(1.98)
Hospital	0.989	0.978 ***	0.980 ***	−0.057 ***	−0.032
	(−0.51)	(−4.51)	(−4.91)	(−7.69)	(−0.33)
Inst. Age	1.003 *	1.000	1.000	0.004 ***	−0.02 ***
	(2.37)	(−0.60)	(−0.10)	(6.47)	(−2.90)
Institution Size	1.005	1.020 ***	1.018 ***	0.066 ***	--
	(1.64)	(24.65)	(25.28)	(56.27)	
Time Eff.	Yes	Yes	Yes	Yes	Yes
Dist. Fixed Eff.	Yes	Yes	Yes	Yes	Yes
Obs. No.	633,680	633,680	633,680	633,680	633,680

Note: Odds ratios are reported for the logit regression, and IRRs are reported in the Poisson and negative binomial regressions. Z-statistics are in parentheses. ***, **, and * represent 1%, 5%, and 10% statistical significance.

**Table 6:** Regression results for matched data.

	(1)	(2)	(3)	(4)	(5)
	Logit	Poisson	NBinomial	OLS	2SLS
Policy Effects					
Point	0.0324 ***	1.419 ***	1.262 ***	0.72 ***	2.32 ***
	(−9.04)	(8.04)	(4.79)	(10.51)	(3.91)
Copay	0.999	0.952 ***	0.959 ***	0.044 ***	−0.006 *
	(−0.01)	(−7.72)	(−6.07)	(4.11)	(−1.66)
IOL		1.427 ***	1.375 ***	3.31 ***	15.84 ***
		(30.88)	(27.65)	(234.33)	(4.65)
Pt Chara.					
Male	1.733 ***	0.986 ***	0.978 ***	0.018 **	--
	(15.20)	(−3.38)	(−5.69)	(3.11)	
Age	1.181 ***	0.988 ***	0.991 ***	−0.008 **	−0.589 ***
	(8.47)	(−5.05)	(−3.59)	(−2.67)	(−4.05)
Income	0.708 ***	1.001	1.001	−0.015 *	0.086
	(−7.66)	(0.15)	(0.26)	(−2.07)	(1.30)
Premium	1.397 ***	0.998	0.998	0.010 *	−0.911 **
	(10.59)	(−0.58)	(−0.52)	(2.07)	(−1.91)
CCI	0.350 ***	1.123 ***	1.112 ***	0.151 ***	0.48 ***
	(−39.56)	(48.23)	(41.74)	(33.91)	(4.71)
Employment					
Public Sector	2.188 ***	0.944 *	0.947 *	−0.48	−0.62 *
	(3.91)	(−2.48)	(−2.33)	(−1.29)	(−1.85)
Private Sector	4.208 ***	0.988	0.986	0.012	−0.50 **
	(19.27)	(−1.46)	(−1.70)	(0.99)	(−3.02)
Farm	7.457 ***	0.929 ***	0.941 ***	0.021	−0.564 **
	(22.63)	(−7.64)	(−6.23)	(1.48)	(−2.68)
Education	2.848 ***	0.991	0.987	0.001	−0.49 *
	(7.25)	(−0.61)	(−0.81)	(0.02)	(−1.90)
Indep. Contract	0.476 ***	0.996	0.996	−0.06 *	0.117
	(−4.40)	(−0.24)	(−0.21)	(−2.05)	(−0.48)
Other	4.329 ***	0.988	0.989	0.013	−0.48 ***
	(18.30)	(−1.40)	(−1.19)	(0.99)	(−2.83)
Institution Ch.					
HHI	1.195 ***	1.024 ***	1.034 ***	0.056 ***	−0.06
	(5.04)	(6.20)	(8.03)	(9.84)	(−1.31)
Med_center	2.182 ***	1.008	1.000	0.083 ***	−0.11 *
	(11.62)	(1.05)	(−0.03)	(6.90)	(−1.59)
Hospital	1.160 *	1.002	0.995	−0.124 ***	−0.003
	(2.01)	(0.23)	(−0.53)	(−9.13)	(−0.12)
Inst. Age	1.032 ***	1.003 ***	1.002 ***	−0.007 ***	−0.004 ***
	(7.42)	(6.75)	(4.20)	(−2.97)	(−2.68)
Institution Size	1.048 **	1.027 ***	1.029 ***	0.048 ***	--
	(3.19)	(19.74)	(19.42)	(23.08)	
Time Eff.	Yes	Yes	Yes	Yes	Yes
Dist. Fixed Eff.	Yes	Yes	Yes	Yes	Yes
Obs. No.	165,764	165,764	165,764	165,764	165,764

Note: ***, **, and * represent 1%, 5%, and 10% statistical significance.

## Data Availability

Data are unavailable due to privacy protections for individual patient records enforced by the Taiwan National Health Insurance Administration.
